# Impact of Lipoprotein(a) on Macrovascular Complications of Diabetes in a Multiethnic Population in the French Amazon

**DOI:** 10.1155/2023/8111521

**Published:** 2023-02-03

**Authors:** Sabrina Dordonne, Mayka Mergeayfabre, Nezha Hafsi, Andre Ntoutoum, Clara Salazar-Cardozo, Olivier Casse, Marianne Hounnou, Antoine Adenis, Jean-Markens Aurelus, Caroline Misslin-Tristch, Jean-François Carod, Bertrand De Toffol, Jean François Lienne, Magalie Demar, Mathieu Nacher, Nadia Sabbah

**Affiliations:** ^1^Department of Endocrinology and Metabolic Diseases, F-97306 Cayenne, French Guiana; ^2^Clinical Investigation Center Antilles French Guiana (CIC INSERM 1424), Cayenne Hospital Center, F-97306 Cayenne, French Guiana; ^3^Department of General Medicine, Ouest French Guianais Hospital Center, F-97397 Saint-Laurent, French Guiana; ^4^Laboratory of Biology, Ouest French Guianais Hospital Center, F-97397 Saint-Laurent, French Guiana; ^5^Department of Neurology, Cayenne Hospital Center, F-97306 Cayenne, French Guiana; ^6^Laboratory of Parasitology-Mycology (LHUPM), Cayenne Hospital Center, F-97306 Cayenne, French Guiana; ^7^EA3593, Amazon Ecosystems and Tropical Diseases, University of Guiana, French Guiana

## Abstract

**Background and Aims:**

In French Guiana, the prevalence of diabetes is around 10%, and cardio and neurovascular pathologies are the first medical cause of early mortality. Lipoprotein(a) (Lp(a)) is described in the literature as a risk factor independent of other cardiovascular risk factors, but there are important interindividual differences, especially according to ethnicity. The objective of this study was to investigate the association of Lp(a) and macrovascular complications in a multiethnic population of patients with diabetes in the French Amazon.

**Materials and Methods:**

Since May 2019, 1243 patients were screened 806 of whom had Lp(a) determination. We compared the prevalence of macrovascular complications in three groups according to Lp(a) concentration: between 0 and 75 mg/mL, between 76 and 300 mg/mL, and >300 mg/mL.

**Results:**

712 patients in the study had type 2 diabetes (88.34% of the sample). A history of hypertension was significantly associated with greater Lp(a) levels. Lp(a) concentration was greater among Creole ethnic groups. No association was found between Lp(a) levels and macrovascular complications in the Lp(a) > 300 mg/mL group.

**Conclusions:**

These results do not replicate findings in mostly Caucasian populations suggesting that the Lp(a) threshold for, or the link with, cardiovascular risk may be different given the predominantly African origin of the French Guianese population. Further studies should study genetic polymorphisms in our population.

## 1. Introduction

Diabetes mellitus, in particular, type 2 diabetes, is a growing public health problem affecting more than 422 million people in the world [1]. Diabetes increases cardiovascular risks with a female predominance [2] independently from other frequently associated risk factors, notably high blood pressure. Cardiovascular complications are indeed the leading cause of death in patients with type 2 diabetes [3]. Diabetes mellitus promotes the formation and progression of atherosclerotic plaques, probably through chronic inflammation and abnormalities in lipid and carbohydrate metabolisms [4]. Lipoprotein(a) (Lp(a)) is a risk factor that is independent from other cardiovascular risk factors; it is genetically determined in an autosomal dominant mode, but there are large intra- and interethnic variations [5, 6]. Some studies, such as the ARIC cohort in the United States [7], have shown that elevated levels of Lp(a) in Caucasians with diabetes or prediabetes were associated with an increased risk of atheromatous cardiovascular disease and that the addition of Lp(a) to traditional risk factors improved the prediction of cardiovascular risk. Lp(a) is a glycoprotein synthesized in the liver from a low-density lipoprotein- (LDL-) like molecule, apolipoprotein B100, which is covalently associated with apolipoprotein(a) via a disulfide bridge [7, 8]. Lp(a) has a high sequence homology with plasminogen, which gives it prothrombotic properties and allows it to compete with plasminogen, thus impeding antifibrinolytic activity [9]. The concentration of Lp(a) is inversely proportional to the number of protein domains that compose it (from 0.01 to 3 g/L) [10]. The small molecular weight isoforms are the most atherogenic and those with the highest concentration, but there is also significant variation related to gene polymorphism that explains some of the differences in circulating concentrations between different populations [5]. According to the European Society of Atherosclerosis (EAS), screening for elevated Lp(a) should be performed in subjects at high cardiovascular risk with premature coronary artery disease, familial hypercholesterolemia, in patients with a family history of coronary artery disease, and in those with recurrent coronary artery disease despite lipid-lowering therapy, which includes a large proportion of patients with diabetes [3]. However, there have been no clear recommendations for the measurement of Lp(a) in the prevention of cardiovascular risk, notably because of the lack of active therapies to reduce Lp(a). Nevertheless, treatments are being evaluated, and recommendations have been formalized, notably in France [11]. In French Guiana, although cardiovascular pathologies are a leading cause of mortality, the epidemiological, clinicobiological, and sociodemographic characteristics of diabetic patients are not well known [12]. In French Guiana, diabetes mellitus is probably underestimated. Its prevalence increased by 57% between 2004 and 2007, and in 2014, it was estimated at 9.3% [13]. The incidence of stroke is twice that of mainland France [14]. In this context, the objective of our study was to investigate the association of Lp(a) and macrovascular complications in a multiethnic population of patients with diabetes mellitus.

## 2. Materials and Methods

This multicenter cross-sectional prospective study was carried out at Cayenne Hospital, within the Endocrinology-Diabetology-Nutrition Department, at Saint-Laurent-du-Maroni Hospital, and in French Guiana.

Overall, 1243 participants with diabetes mellitus were preincluded in the CODIAM cohort, and 806 who benefitted from Lp(a) measurement were retained. Diabetes was defined as an increase in fasting plasma blood glucose greater than or equal to 126 mg/dL (or 7 mmol/L) on two occasions and/or a blood glucose level taken at any time of the day greater than or equal to 200 mg/dL (or 11 mmol/L) on two occasions. We followed the methods of Linière et al. [15].

### 2.1. Inclusion Criteria

We included patients who were at least 18 years old at the time of the study. The patients had a definite diagnosis of diabetes mellitus. All included patients signed a written consent.

### 2.2. Exclusion Criteria

We excluded patients who did not benefit from Lp(a) measurement and those who were minors, under guardianship, who refused to participate or did not sign the consent form, who were deprived of liberty, who presented acute life-threatening situations, who had gestational diabetes, or who were seen during pregnancy and *postpartum* (less than 6 months).

### 2.3. Data Collection

Patients were included (first blood measurement V1) during consultations at the Endocrinology-Diabetology-Nutrition Department of Cayenne Hospital and at the General Medicine Department of Saint-Laurent-du-Maroni Hospital between May 2019 and December 2021. They were also recruited during hospitalizations in Medical Departments of Cayenne Hospital. Demographic characteristics, medical history (diabetes complications), examination data, and biological results of the patient were collected. Systolic and diastolic blood pressures (TAS and TAD) were measured on the day of the inclusion visit, and the history of arterial hypertension was recorded. The other variables studied were age, sex, mother language, body mass index (BMI), abdominal circumference, plasma Lp(a) concentration, plasma LDL cholesterol concentration, glycated hemoglobin, creatinine level, creatinine clearance, history of angina (angina pectoris was noted only if a diagnosis of certainty was made after hospitalization with electrocardiographic confirmation, cardiac ultrasound and stress test and/or coronary CT scan, or coronary angiography (more rarely stress MRI)), history of obliterating arteriopathy of the lower limbs, history of cerebrovascular accident, and history of transient ischemic attack.

The group with atypical diabetes corresponds to secondary diabetes due to pancreatic damage or African diabetes (ketosis prone diabetes).

Patients with diabetes were classified into three categories based on Lp(a) levels: 0-75 mg/mL, 76-300 mg/mL, and>300 mg/mL.

The Lp(a) assays were standardized and carried out at the laboratories of Cayenne and Saint-Laurent-du-Maroni Hospital and expressed in milligrams per milliliter (mg/mL).

### 2.4. Statistical Analysis

Statistical analysis was performed using STATA software® (STATA-CORP®, College Station, Texas, USA). The descriptive analysis consisted in an epidemiological, biological, and clinical description of the population of diabetic patients who had at least one Lp(a) assay. Categorical variables were expressed as frequencies and percentages while continuous variables relied on mean and standard deviation. Parametric or nonparametric tests were done for comparison of quantitative variables between groups, as appropriate. Percentages were compared using the chi-square test. The significance level was 5%.

### 2.5. Regulatory and Ethical Aspects

All included patients were informed of the anonymous use of their data for the research. In accordance with the French Data Protection Act and the General Data Protection Regulation, the data processing was subject to a data protection impact analysis, an entry in the hospital's data processing register, and a declaration of compliance MR003. The protocol was approved by the Comité de Protection des Personnes Sud-Est de Clermont-Ferrand (Nos ref: 2020/CE 05). All patients provided written informed consent for participation and publication of anonymized study results.

## 3. Results

### 3.1. General Characteristics of the Study Population

1243 patients were preincluded in the CODIAM cohort between 2019 and 2021. Of these, 806 patients had an Lp(a) assay at their first V1 inclusion visit and were retained for the present study. The Guianese population in the study was predominantly of African descent with notably Creole mother language (Table 1). Over half (54.12%) of the women had Lp(a) > 300 mg/mL. There was no significant difference in Lp(a) levels between genders.

712 patients had type 2 diabetes (88.34%), 48 patients had type 1 diabetes (5.95%), 11 patients had latent autoimmune diabetes in adult diabetes (1.36%), 2 patients had MODY diabetes (0.25%), and 33 patients had atypical diabetes (4.09%).

Clinical and biological characteristics are presented in Table 2. There was a nonsignificant trend for greater Lp(a) levels in older patients: 55.0 years for Lp(a) between 0 and 75 mg/mL, 56.3 years for Lp(a) between 76 and 300 mg/mL, and 58 years for Lp(a) > 300 mg/mL.

We found a significant association between Lp(a) concentration and hypertension (Table 3).

### 3.2. Macrovascular Complications in Patients with Diabetes according to Lp(a) Level

Table 4 shows the absence of relation between Lp(a) concentration and cardiovascular complications. We did not find any association between the Lp(a) level and a history of *angina pectoris*, stroke, or lower limb arteriopathy (and no difference was found for levels above 500 mg/L; Table 5).

There was a nonsignificant trend for greater concentrations of Lp(a) among patients receiving statins relative to those who did not, respectively (median = 319 (IQR = 117 − 679) vs. 271 (IQR = 75 − 633), *p* = 0.10).

## 4. Discussion

In contrast to the majority of studies evaluating the association between the risk of coronary events in patients with type 2 diabetes and Lp(a) levels [6], our study did not report a significant association between the presence of a history of angina and high Lp(a) levels. This lack of association is presumably related to the composition of our study population, which was essentially of African ancestry. Indeed, the association between Lp(a) levels and cardiovascular complications has been mainly established in European ethnic groups [16]. Emdin et al. via their study of several large cohorts showed that Lp(a) was significantly associated with the occurrence of coronary heart disease (odds ratio (OR) = 1.28 (1.16-1.41)), stroke (OR = 1.14 (1.07-1.21)), peripheral arterial disease (OR = 1.22 (1.11-1.34)), and abdominal aortic aneurysm in European populations. By contrast, this association was not replicated in populations of African ancestry for coronary heart disease (OR = 1.11 (0.99-1.24)) and cerebrovascular disease (OR = 1.06 (0.99-1.14)); however, similar associations were found for peripheral arterial disease (OR = 1.16 (1.01-1.33)) and abdominal aortic aneurysm (OR = 1.34 (1.11-1.62)) [17].

Waldeyer et al. confirmed through their meta-analysis that Lp(a) was a marker of cardiovascular risk in the European population with an increased risk for Lp(a) levels above the 66^th^ percentile [18]. Cardiovascular diseases remain the leading cause of death in the majority of patients with diabetes mellitus. Although no significant relation was found between Lp(a) levels and history of angina, stroke, or obliterative arterial disease of the lower limbs in our study, the proportion of patients with the aforementioned histories was systematically higher for the highest Lp(a) concentrations.

For the first time, our study allowed to evaluate the relationship between Lp(a) levels and macrovascular complications in patients with mostly type 2 diabetes in a multiethnic population in the French Amazon. Most patients had high Lp(a) levels, above 300 mg/mL, and levels were generally greater in groups with a mother language reflecting African ancestry. These results are in agreement with other studies that found that ethnic groups of African origin had higher concentrations of Lp(a) than individuals of European origin. Indeed, black populations had Lp(a) levels twice as high as the Caucasian populations [19]. Similarly, Indians had higher Lp(a) levels than the Chinese, but their Lp(a) levels seemed intermediate between those of blacks and whites [20]. Lp(a) level is essentially explained by the molecular weight of apolipoprotein (a) (a component of Lp(a)) which can vary between individuals and is genetically determined; this is inversely proportional to the domains which comprise it (the type 2 kringles IV) [21]. Apolipoprotein (a) hence varies considerably in size and molecular weight (between 300 and 800 kDa). Individuals with small molecular weight apolipoprotein (a) isoforms have higher plasma concentrations of Lp(a) and are described as having the greatest atherogenic potential [22]. This seems at odds with our findings where Lp(a) concentrations were high but without association with cardiovascular complications. This conundrum cannot be resolved with the current data; presumably, the in depth description of human genetic polymorphisms and of the molecular details of Lp(a) in French Guiana would help understand why.

It should be noted that our population was relatively young (mean age = 55 years), and it is well known that cardiovascular complications in diabetes are associated with the duration of the disease (largely correlated with age), the presence of microvascular complications, and the number of nontargeted risk factors [23]. Here, we found a nonsignificant trend of increasing Lp(a) levels with age. This hypothesis has already been reported in other studies where specific cut-off values for Lp(a) levels had been proposed in relation to age [24]. Slunga and colleagues found a weak but significant relationship between age and increased Lp(a) levels in Sweden [25]. However, there are conflicting studies [26]. Although the results vary from study to study, it is increasingly accepted that Lp(a) is characterized by a very wide range of plasma concentrations (0.01 to >3 g/L; ≈25 to 750 nmol/L) that are primarily influenced by genetic factors, but not by age, sex, or lifestyle [11]. Although many population-based studies have indicated no difference in Lp(a) concentrations between men and women, others indicate higher Lp(a) levels in women, as in our study where most had Lp(a) concentrations > 300 mg/mL. Nevertheless, there are potential confounding factors such as ethnicity and menopausal status [26]. All patients in the study were overweight or obese with a mean BMI of 31.1. This result is in agreement with a recent study in the French Guianese population studying the specificities of the diabetic population in French Guiana [13]. On the other hand, abdominal circumference was found to be lower in patients with Lp(a) > 300 mg/mL compared with those with lower Lp(a) concentrations, and to our knowledge, there is no literature describing this result. One study in the literature in 1997 looked at the effect of adipose mass loss, particularly visceral fat, on Lp(a) concentration and found no relationship [27].

A history of hypertension was significantly associated with Lp(a) levels in our diabetic patient population with 53.67% of patients with Lp(a) > 300 mg/mL. This relation had also been mentioned by Langsted et al. and Yeang et al. in their study concerning the elevation of Lp(a) levels and the risk of ischemic stroke, who had found a progressive increase in the absolute risk of ischemic stroke with increasing Lp(a) levels across all categories of sex, age, smoking, and hypertension [28, 29]. The presence of hypertension in diabetic patients significantly increases the risk of macrovascular and microvascular complications. The pathophysiology of arterial hypertension is explained by arterial aging resulting in thickening and stiffening of the arterial wall. The various mechanisms involved in the process of arteriosclerosis are complex, and some of them are common to those that lead to atherosclerosis lesions, namely, dysfunction of the endothelium, remodeling of the parietal extracellular matrix, and calcification of the media [30]. An in vitro study performed on rabbit renal arteries showed that Lp(a) alters, in a dose-dependent manner, the dilatation properties of the endothelium via oxygen production and nitric oxide inactivation. Fytili et al. showed significantly higher Lp(a) values in hemodialysis patients with hypertension than in those without hypertension. They emphasized that hypertension may play an important role in serum Lp(a) titers in end-stage renal disease patients on dialysis [31]. Finally, as we found no significant difference in Lp(a) concentration between those with or without statins, a potential confounding effect by statin use [32] and its impact on Lp(a) concentration did not seem likely to explain the lack of association between Lp(a) and cardiovascular diseases in French Guiana.

Among the study limitations, the inference of African ancestry through mother language is imperfect and reflects the fact that ethnic statistics are not authorized in France. The measurements during the inclusion visit only allow a retrospective analysis of cardiovascular events. Despite these limitations, the present results suggest that Lp(a) may indeed be of limited use for the clinician in our multiethnic population where the great heterogeneity of Lp(a) structure and concentrations may trump any effort to define a single threshold that makes sense across populations.

## 5. Conclusion

Although a significant association between hypertension and elevated Lp(a) concentrations was found, our study did not find an association between Lp(a) levels and macrovascular complications. These results are partly due to the predominant African ancestry of the Guianese population. Thus, the Lp(a) threshold(s) corresponding to cardiovascular risk maybe different given the essentially African origin of the French Guianese population (a crude simplification of the local ethnic mix), and this raises the question of the indications for treatment of Lp(a) in different ethnic groups. Overall, given the lack of obvious association of Lp(a) with cardiovascular complications, its use as a predictor of risk seems currently unwarranted in French Guiana. In our heterogeneous population, Lp(a) presumably represents a heterogeneous group of molecules, a situation that makes it difficult to study simple associations when overlooking this problem. Genetic studies in the French Guianese population would allow the analysis of local polymorphisms and may yield more useful information.

## Figures and Tables

**Table 1:** Distribution of gender and mother tongue according to lipoprotein(a) categories.

Variables	Lp(a) categories			*p* value	Overall (%)
	0-75 mg/mL (*N* = 198)	76-300 mg/mL (*N* = 220)	>300 mg/mL (*N* = 388)		
Mother tongue				0.00	
French (%)	41 (20.71)	25 (11.36)	37 (9.54)		103 (12.78)
Guianese Creole (%)	39 (19.70)	49 (22.27)	105 (27.06)		193 (23.95)
Haitian Creole (%)	21 (10.61)	72 (32.73)	125 (32.22)		218 (27.05)
Antilles Creole (%)	10 (5.05)	13 (5.91)	27 (6.96)		50 (6.20)
Portuguese (%)	36 (18.18)	15 (6.82)	18 (4.64)		69 (8.56)
Spanish (%)	7 (3.54)	9 (4.09)	8 (2.06)		24 (2.98)
Dutch (%)	1 (0.51)	2 (0.91)	6 (1.55)		9 (1.12)
English (%)	8 (4.04)	7 (3.18)	25 (6.44)		40 (4.96)
Other (%)	35 (17.68)	28 (12.73)	37 (9.54)		100 (12.41)
Sex				0.92	
Men	94 (47.47)	101 (45.91)	178 (45.88)		373 (46.28)
Women	104 (52.53)	119 (54.09)	210 (54.12)		433 (53.72)

**Table 2:** Description of the study population according to Lp(a) categories.

Variables Lp(a)	Lp(a) categories			Overall (%)
	0-75 mg/mL (*N* = 198)	76-300 mg/mL (*N* = 220)	>300 mg/mL (*N* = 388)	
Weight (kg) Mean (SD)	83.8 ± (18.35)	84.2 ± (20.66)	84.8 ± (19.5)	703
Age Mean (SD)	55.00 ± (12.39)	56.30 ± (13.26)	57.98 ± (12.63)	712
BMI (kg/m^2^) Mean (SD)	31.32 ± (5.88)	31.29 ± (7.63)	30.99 ± (6.57)	700
Systolic blood pressure (mmHg) Mean (SD)	134 ± (16.11)	142 ± (17.43)^∗∗^	143 ± (19.60)^∗∗∗^	704
Diastolic blood pressure (mmHg) Mean (SD)	78 ± (11.18)	80 ± (12.03)	80 ± (14.36)	704
Waist size (cm)	106.2 ± (14.02)	107.6 ± (16.64)	102.8 ± (14.71)	257
Creatinine (*μ*mol/L) Mean (Q1; Q3)	75.3 [54.5; 80.0]	84.3 [53.0; 89.0]	87.8^∗^ [61.0; 93.0]	696
Creatinine clearance (mL/min/1.73 m2) Mean (Q1; Q3)	104.3 [87.0; 124]	102.8 [88.0; 122]	94.5^∗∗^ [77.0; 117]	574
Glycated hemoglobin (%) Mean (SD)	8.7 ± (2.20)	8.7 ± (2.06)	8.6 ± (2.06)	685
LDL-c (g/L) Mean (SD)	2.55 ± (0.93)	2.46 ± (0.88)	2.66 ± (0.95)	649

Comparison between Lp(a) 0-75 mg/mL and Lp(a) 76-300 mg/mL. Comparison between Lp(a) 0-75 mg/mL and Lp(a) > 300 mg/mL. Significance level: ^∗^*p* < 0.05, ^∗∗^*p* < 0.01, and ^∗∗∗^*p* < 0.001.

**Table 3:** Distribution of the number (No.) of patients by history of hypertension by lipoprotein(a) level.

	Lp(a) 0-75 mg/mL	Lp(a) 76-300 mg/mL	Lp(a) >300 mg/mL	*p* value	Total
History of high blood pressure		No. (%)			
Yes	99 (61.88)	122 (68.16)	256 (78.05)	0.001	477 (71.51)
No	61 (38.13)	56 (31.28)	72 (21.95)		189 (28.34)
Unknown	0 (0.00)	1 (0.56)	0 (0.00)		1 (0.15)

**Table 4:** Distribution of the number (No.) of patients by diabetes-related macrovascular complications according to lipoprotein(a) level.

	Lp(a) 0-75 mg/mL	Lp(a) 76-300 mg/mL	Lp(a) >300 mg/mL	*p* value	Total
Macrovascular complications		No. (%)			
Angina					
Yes	6 (4.96)	4 (2.80)	12 (4.94)	0.30	22 (4.34)
No	110 (90.91)	138 (96.50)	226 (93.00)		474 (93.49)
Unknown	5 (4.13)	1 (0.70)	5 (2.06)		11 (2.17)
Transient ischemic attack					
Yes	10 (8.20)	1 (0.70)	10 (4.13)	0.050	21 (4.15)
No	110 (90.16)	138 (97.18)	226 (93.39)		474 (93.68)
Unknown	2 (1.64)	3 (2.11)	6 (2.48)		11 (2.17)
Stroke					
Yes	15 (12.30)	8 (5.63)	21 (8.64)	0.30	44 (8.68)
No	106 (86.89)	134 (94.37)	221 (90.95)		461 (90.93)
Unknown	1 (0.82)	0 (0.00)	1 (0.41)		2 (0.39)
Arteriopathy					
Yes	12 (6.06)	8 (3.64)	28 (7.25)	0.38	48 (5.97)
No	160 (80.81)	177 (80.45)	309 (80.05)		646 (80.35)
Unknown	26 (13.13)	35 (15.91)	49 (12.69)		110 (13.68)

**Table 5:** Distribution of the number (No.) of patients with diabetes-related macrovascular complications according to Lp(a) level: analysis at the 500 mg/mL threshold.

	Lpacat 0-500 mg/mL	Lpacat > 500 mg/mL	*p* value	Total
Macrovascular complications		No. (%)		
Angina pectoris				
Yes	14 (4.09)	8 (4.85)	0.231	22 (4.34)
No	318 (92.98)	156 (94.55)		474 (93.49)
Unknown	10 (2.92)	1 (0.61)		11 (2.17)
AIT				
Yes	15 (4.39)	1 (3.66)	0.607	21 (4.15)
No	321 (93.86)	153 (93.29)		474 (93.68)
Unknown	6 (1.75)	5 (3.05)		11 (2.17)
Stroke				
Yes	27 (7.89)	17 (10.30)	0.573	44 (8.68)
No	314 (91.81)	147(89.09)		461 (90.93)
Unknown	1 (0.29)	0 (0.61)		2 (0.39)
Arteriopathy				
Yes	28 (5.13)	20 (7.73)	0.288	48 (5.97)
No	440 (80.59)	206 (79.84)		646 (80.35)
Unknown	78 (14.29)	32 (12.40)		110 (13.68)

## Data Availability

Data will be available on request by mail through the corresponding author.

## Abbreviations

Lp(a): Lipoprotein(a) in milligrams per milliliter (mg/mL)
LDL: Low-intensity lipoprotein
HDL-c: HDL cholesterol
LDL-c: LDL cholesterol
BMI: Body mass index
SBP: Systolic blood pressure
DBP: Diastolic blood pressure
TIA: Transient ischemic attack
HbA1c: Glycated hemoglobin.
